# Report of Positive Placental Swabs for SARS-CoV-2 in an Asymptomatic Pregnant Woman with COVID-19

**DOI:** 10.3390/medicina56060306

**Published:** 2020-06-22

**Authors:** Antonella Ferraiolo, Fabio Barra, Chiara Kratochwila, Michele Paudice, Valerio Gaetano Vellone, Elisabetta Godano, Serena Varesano, Giovanni Noberasco, Simone Ferrero, Cesare Arioni

**Affiliations:** 1 Unit of Obstetrics and Gynaecology, IRCCS Ospedale Policlinico San Martino, Largo R. Benzi 10, 16132 Genova, Italy; antonellaferraiolo@hotmail.it; 2 Academic Unit of Obstetrics and Gynaecology, IRCCS Ospedale Policlinico San Martino, Largo R. Benzi 10, 16132 Genova, Italy; chiara.kratochwila@gmail.com (C.K.); simoneferrero@me.com (S.F.); 3 Department of Surgical and Diagnostic Sciences, IRCCS Ospedale Policlinico San Martino, University of Genova, 16132 Genoa, Italy; paux91@live.it (M.P.); vgvellone@gmail.com (V.G.V.); 4 Neonatology Unit, University of Genoa, IRCCS Ospedale Policlinico San Martino, 16132 Genoa, Italy; elisabetta.godano@gmail.com (E.G.); cesare.arioni@hsanmartino.it (C.A.); 5 Department of Health Sciences, University of Genoa, Via Pastore 1, 16132 Genoa, Italy; sere_varesano@hotmail.it (S.V.); noberasco.giovanni@gmail.com (G.N.)

**Keywords:** COVID-19, SARS-CoV-2, pregnancy, placenta, rhino-pharyngeal swab, placental swab, cesarean section

## Abstract

Currently, limited data on maternal and neonatal outcomes of pregnant women with infection and pneumonia related to SARS coronavirus 2 (SARS-CoV-2) are available. Our report aims to describe a case of placental swabs positive for the molecular research on severe acute respiratory syndrome coronavirus 2 (SARS-CoV-2 RNA in an asymptomatic woman with positive rhino-pharyngeal swab for SARS-CoV-2 who underwent an urgent cesarean section in our obstetrics unit. Sample collection, processing, and laboratory testing were conducted in accordance with the World Health Organization (WHO) guidance. In the next months, conclusive data on obstetrical outcomes concerning the gestational age and pregnancy comorbidity as well as the eventual maternal–fetal transmission are needed.

## 1. Introduction

The outbreak of coronavirus disease 2019 (COVID-19) occurred in Wuhan (Hubei Province, China) at the end of December 2019, and it has been declared a pandemic in March 2020 by the World Health Organization (WHO). As of 20 June, 2020, COVID-19 has caused 8,666,697 confirmed cases and 460,066 deaths globally, including 238,011 confirmed cases and 34,561 deaths in Italy [1]. However, the real prevalence and mortality rates of COVID-19 are still unknown due to the large presence of asymptomatic and mild symptomatic patients. 

COVID-19 is etiologically caused by SARS coronavirus 2 (SARS-CoV-2), a virus closely related to the SARS virus [2]. In COVID-19, the period between the infection and onset of symptoms is approximately 14 days. The disease can be asymptomatic or presenting with a wide range of symptoms, including fever, cough, diarrhea, fatigue, and mild pneumonia; even acute respiratory distress syndrome (ARDS) with multiorgan failure and intravascular disseminated coagulopathy may occur. Nevertheless, the severity of the infection depends on a variety of factors, such as age and comorbidity of patients [3,4]. The mode of transmission of SARS-CoV-2 infection is based on release of droplets and aerosols [2].

Pregnant women are more susceptible to respiratory infections due to the physiologically adaptive changes of the respiratory tract, such as diaphragm elevation, increased oxygen consumption, and edema of the respiratory tract mucosa [5]; moreover, high concentrations of estrogens cause congestion and excessive secretion of airway epithelial cells [6,7]. The association between adverse obstetrical–neonatal outcomes and physiopathological changes related to respiratory viral infection has been reported in severe acute respiratory syndrome (SARS) and the Middle East respiratory syndrome (MERS) [8]; nevertheless, it has been suggested that the clinical presentation of COVID-19 in pregnant women tends to be similar to that of the normal population [4]. 

There is no evidence from the studies of the current literature about COVID-19 maternal–fetal transmission during pregnancy and, in particular, in the third trimester of pregnancy, or during postpartum breastfeeding [9]; however, further studies will draw a conclusion on this emergent topic, which not only has a significant public health impact but also represents a real issue for the management of pregnant women with suspected or confirmed COVID-19 [7]. 

This report aims to describe a case of placental swabs positive for SARS-CoV-2 RNA obtained from an asymptomatic woman in the third trimester of pregnancy with positive rhino-pharyngeal swab, who underwent urgent cesarean section in our center.

## 2. Case Report

A 30-year-old Italian Gravida 1 Para 0-0-0-0 at 38 3/7 weeks was hospitalized the day before undergoing an elective cesarean section for breech presentation, for whom external cephalic version has been unsuccessful. In the anamnesis, the patient did not report any previous disease or pregnancy-related illness, and she had regular ultrasonographic scans in the first, second, and third trimesters of pregnancy. Combined test in the first semester of pregnancy showed a low risk for Down syndrome, and the morphological ultrasonographic scan in the second semester of pregnancy was regular. In the third trimester ultrasonographic scan performed at 31 4/7 weeks, the fetus had normal velocimetric values in the umbilical and middle cerebral arteries and a regular amount of amniotic fluid (amniotic fluid index (AFI), 15.5 mm); the estimated fetal weight was 2122 g (86.4 percentile). 

Since April 2020, the obstetric unit of our hospital (IRCCS Ospedale Policlinico San Martino, Genoa, Italy) has instituted a protocol providing the performance of rhino-pharyngeal swab to investigate the presence of SARS-CoV-2 RNA by Allplex™ 2019 n-CoV assay multiplex quantitative real-time PCR (qRT-PCR) (Seegene Inc., Seoul, Korea) and anti-SARS-CoV-2 immunoglobins by MAGLUMI™ 2019-nCoV IgM and IgG assays (Snibe, Shenzhen, China). Nasopharyngeal samples were taken by Universal Transport Medium® (UTM-RT; Copan spa, Brescia, Italy).

After performing the swab and serology for SARS-CoV-2, the woman was admitted to our unit in a single room to avoid eventual interpersonal contact because of her unknown infective status. At admission and in the previous 15 days, the pregnant woman did not have fever and respiratory and gastrointestinal symptoms, myalgia, malaise, ageusia, or anosmia. Moreover, she declared not having had contact with certain or suspected COVID-19 cases in the previous 15 days. Her tympanic temperature was 36.2 °C (97.2 °F), blood pressure was 123/83 mmHg, heart rate (HR) was 83 bpm, and peripheral capillary oxygen saturation was 99% in air. Cardiotocography (CTG) showed normoactive reassuring fetal parameters (short-term variability (STV) 12 bpm; baseline fetal HR, 131 bpm; presence of 11 fetal HR accelerations and 0 fetal HR deceleration in 34.2 min of registration -category 1 according to ACOG (American College of Obstetricians and Gynecologists) CTG intrapartum classification) [10]. The patient perceived regular active fetal movements and did not experience uterine contractions. The obstetric visit reported a Bishop score of 2 (posterior closed cervix with soft consistency; 0% effacement; extra-pelvic cephalic presentation). The ultrasound examination showed regular fetal HR and presence of fetal active movement. The placenta had a regular anterior uterine insertion with a grade 2 maturity score [11]. The AFI was 16.5 mm, and the fetal umbilical artery velocimetry was regular (pulsatility index: 0.79; 37.6 percentile). The patient showed moderate anemia (hemoglobin 9.2 g/L; 12.0–16.0 g/L). White blood count (WBC), platelets, hepatic, and renal function had regular values. 

After 8 h of admission, painful irregular uterine activity occurred. The CTG showed normoactive fetal heart rate (presence of 3 uterine contractions every 10 min; short-term STV of 14 bpm, baseline fetal HR of 145 bpm, presence of 9 fetal HR accelerations and 0 fetal HR deceleration in 25.6 min of registration—category 1 according to ACOG cardiotocography intrapartum classification [12,13]). The obstetric examination reported a Bishop score of 6 (intermediate cervix with soft consistency and 3 cm of dilatation; 30% effacement; cephalic presentation, −3). Because of the status of initial labor and the concomitant obstetric relative contraindication to vaginal delivery, the clinical decision was to proceed with performing an urgent cesarean section. As the results of the rhino-pharyngeal swab and serology for SARS-CoV-2 were not yet available, the protocol foreseen for suspected cases of COVID-19 undergoing surgical operative procedures was applied: The cesarean section was performed in a dedicated surgical room; surgeons, anesthesiologists, and the other hospital staff followed sterile dressing and undressing procedure with personal protective equipment. Sample collection, processing, and laboratory testing were collected in accordance with the World Health Organization (WHO) guidance [14].

Epidural anesthesia and caesarian section were performed without complications; amniotic fluid appeared clear and normal in quantity. The newborn was male with an Apgar score of 9 at the first minute and 10 at the fifth and tenth post-birth minutes; his weight was 3310 g. The placenta did not show macroscopic abnormalities and was sent for definitive analysis performed by a dedicated pathological team to placental disease. In the surgery room, a midwife performed three placental swabs from the amniotic surface after clearing the surface of maternal blood in proximity to the umbilical cord and to two opposite peripheral margins. Placental samples were taken by UTM-RT swabs (Copan spa, Brescia, Italy). An additional technical replicate was performed for each placental sample (Supplementary Figure S1).

Preventively, the newborn was separated from the mother and admitted to the neonatological unit, until the definitive results of the rhino-pharyngeal swab of the woman were available. A rhino-pharyngeal swab was also performed in the newborn.

**Figure 1:**
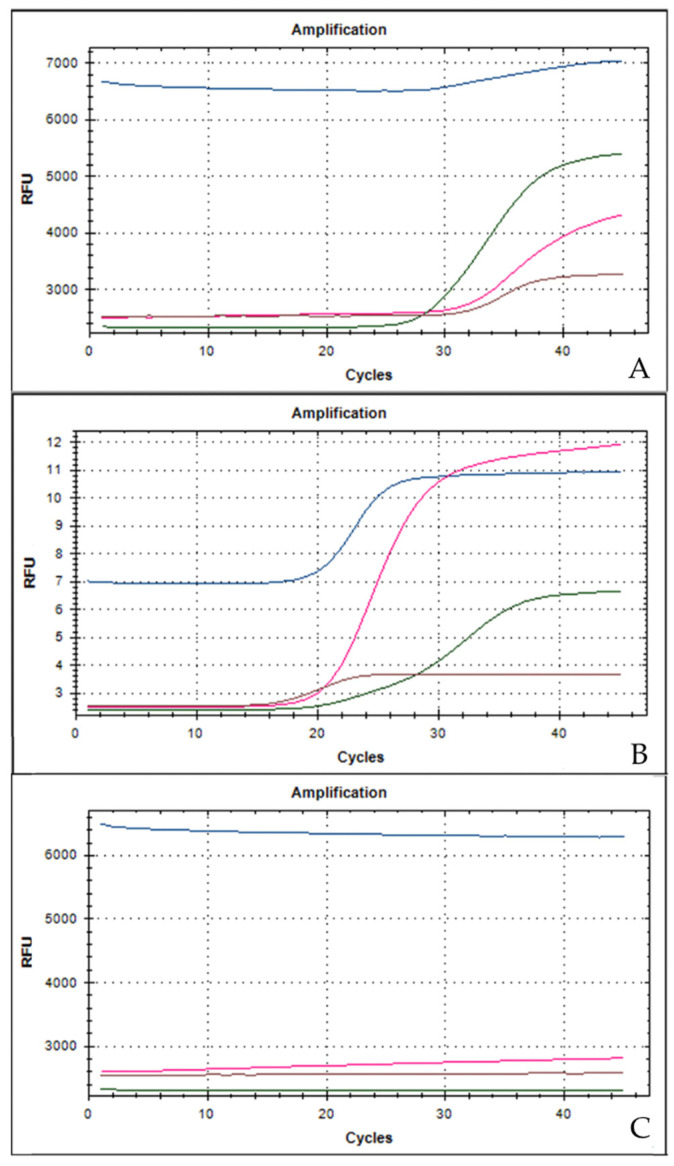
Reverse-transcriptase quantitative PCR amplification curves and high-resolution melting curves of the amplification products. Three target genes of SARS-CoV-2 are amplified in a single tube. The assay is designed to detect RdRP (pink line) and N (brown line) genes specific for SARS-CoV-2, E gene (blue line) for all sarbecoviruses, including SARS-CoV-2 and a process control (green line). (**A**) The placental swab positive for SARS-CoV-2 (sample obtained in the placental fetal surface proximity to the umbilical cord); (**B**) positive control for SARS-CoV-2; (**C**) negative control for SARS-CoV-2.

Only after the conclusion of the surgical procedure did the definitive results of the maternal rhino-pharyngeal swab show positivity for SARS-CoV-2 RNA; the maternal serology showed positive anti-SARS-CoV-2 IgG and negative anti-SARS-CoV-2 IgM. One hour after the surgical procedure, the vital parameters of the woman were stable: The body temperature was 36.6 °C (97.7 °F), blood pressure was 115/80 mmHg, HR was 65 bpm, and SpO2 was 100% in air. White blood count (WBC), and renal and hepatic functions were normal; only C-reactive protein (CRP) and interleukin-6 (IL-6) were above the upper limits of normality (92.3 mg/L (0.0–5.0 mg/L) and 30.2 ng/L (<3.4 ng/L), respectively); the study of antigens for Legionella and Pneumococcus in the urine was negative. Due to optimal peripheral capillary oxygen saturation in air, chest X-ray and computed tomography (CT) were not performed. The day following the surgical procedure, an infective consultation did not suggest the administration of either antiviral or anti-inflammatory drugs due to the asymptomatic clinical presentation, the normal vital parameters, and blood test. The woman voluntarily decided to inhibit lactation onset by cabergoline (0.5 mg BID oral administration). Two days after the surgical procedure, the placental swabs were positive for SARS-CoV-2 RNA (Figure 1); the same day, a second maternal rhino-pharyngeal swab for SARS-CoV-2 confirmed the viral IgG positivity. 

**Figure 2:**
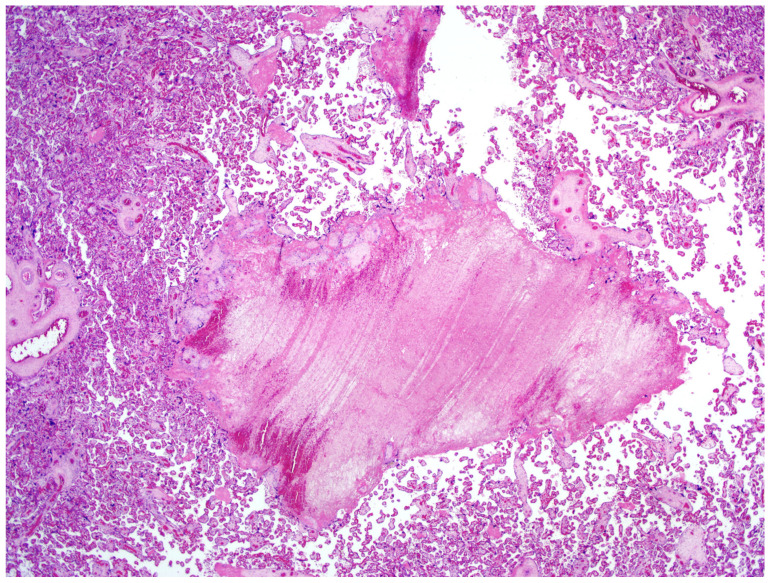
Placenta at microscopy. Hematoxylin–eosin (magnification 20×). Organizing intervillous hemorrhage can be visualized.

The definitive histological analysis of the placenta did not describe substantial macroscopic alterations, except for mild subchorionic deposition of fibrin and for the presence of a single ischemic area in the thickness of the chorionic disc (0.8 cm of maximum diameter). The amniochorial membranes were smooth and characterized by only focal hemorrhages. The three vessels constituting the umbilical cord was slightly hyperspiralized. Microscopically, neither inflammation of membranes nor funisitis were present. The villous tree showed signs of delayed maturation. The terminal villi were characterized by capillary congestion and focal microchorangiosis. Finally, moderate deposition of fibrin, appearance of villous agglutination, and multiple organizing intervillous hemorrhages were also observed (Figure 2). No substantial differences were found in the proximity of the placental sites, in which the placental swabs were performed.

Following the infective consultation and in relation to the obstetrical clinical well-being and regular lab parameters of the woman, we signed the patient off after three days after the caesarean section. Oral iron supplementation and indication to respect social isolation for 20 days were suggested. 

At the 30-day follow-up, the woman and neonate were asymptomatic; a maternal rhino-pharyngeal swab for SARS-CoV-2 confirmed again the viral IgG positivity, whereas a neonatal rhino-pharyngeal swab confirmed IgM and IgG negativity.

The newborn continued being asymptomatic in the neonatology care unit, undergoing three rhino-pharyngeal swabs for the presence of SARS-CoV-2 RNA. The first swab was performed immediately after birth, whereas the other two were done 24 h after the first. Although the first neonatal swab had an inconclusive result, the two others had negative results. At 10 days of life, anti-SARS-CoV-2 serology was performed with negative results for both IgM and IgG. Routinely, neonatal tests, such as otoacoustic emission, direct Coombs test, and red reflex, were normal. Metabolic screening is still in progress. The baby was discharged after 21 days.

## 3. Discussion

No data on the optimal management of pregnant women with suspected or confirmed COVID-19 are present in literature. Pregnant women seem to be particularly susceptible to respiratory pathogens and severe pneumonia due to the anatomical structure of the respiratory system changes during pregnancy [5]; thus, virus transmitted by droplets and aerosols is more easily inhaled by pregnant women. Furthermore, angiotensin-converting enzyme (ACE)-2 has been reported to be highly increased during pregnancy, and this may enhance the susceptibility to SARS-CoV-2 [15]. 

In general, vertical infective transmission may include transmission via germ cells or placental blood during pregnancy, through the birth canal during labor and delivery, and during postpartum breastfeeding [16]. Preliminary data from the current literature have not demonstrated vertical transmission in COVID-19 [17], similarly to severe acute respiratory syndrome (SARS) and Middle East respiratory syndrome (MERS), both caused by coronaviruses [7,18].

This is a report of positive placental swabs for SARS-CoV-2 in an asymptomatic pregnant woman with a positive rhino-pharyngeal swab for SARS-CoV-2 undergoing cesarean section. At the admission and during the hospitalization, the woman remained completely asymptomatic, not presenting fever and/or respiratory and gastrointestinal symptoms; the laboratory examinations did not display suggestive characteristics of COVID-19 syndrome, previously described by other authors [19,20,21]. Nevertheless, because of the absence of the woman’s rhino-pharyngeal swab result, the pregnant patient was managed as suspected for COVID-19, and the cesarean section was performed in a dedicated surgery room. Sterile dressing and undressing procedures were adopted using personal protective equipment, following the guidance disposed from the European Center for Disease Prevention and Control [10], according to our hospital protocol. Having performed the placental swabs in the surgery room immediately after caesarean section in a sterile environment, we are confident of a low risk of sample contamination, which, nevertheless, cannot be completely be excluded. 

The histological examination seems to evidence placental alterations which may be caused by an inflammatory status. In fact, a dedicated pathological team described the presence of intervillous hemorrhage. Indeed, several causes may determine fetal capillary rupture, finally determining variable-sized intervillous hemorrhages and a close contact between maternal and fetal blood [22]. At microscopic analysis, moderate fibrin deposition was observed, even considering that the mother was asymptomatic and had an optimal peripheral capillary oxygen saturation. All these pathological findings together with considering the positive qPCR in placental samples and the maternal positive IgG for SARS-CoV2 cannot exclude the hypothesis that COVID-19 can have short- and middle-term physiopathological consequences on placental issue. To support our consideration, in a previous study, the placental examination of pregnant women affected by severe acute respiratory syndrome (SARS) showed an increased amount of subchorionic fibrin deposition, probably secondary to maternal hypoxia [23]. Recently, a USA retrospective analysis evaluated 32 pregnant patients with COVID-19 who delivered; in 11 of them, placental or membrane swabs were performed. Three swabs returned positive results for SARS-CoV-2; notably, all women had severe to critical COVID-19 infection at time of delivery. None of the infants tested had positive swabs for SARS-CoV-2 on days 1 to 5 of life, and none demonstrated symptoms of COVID-19. Given the mixing of maternal and fetal fluid and tissues at the time of delivery, the authors underscored how the detected SARS-CoV-2 RNA may have an unclear origin [24]. In another Italian case series, SARS-CoV-2 RNA was found on the fetal side of the placenta in two cases of mothers infected by COVID-19 and with neonates also positive for the virus at birth. The placentas of these two women who delivered neonates with SARS-CoV-2-positive rhino-pharyngeal swabs showed chronic intervillositis, with presence of macrophages, both in the intervillous and villous spaces [25]. Different from our study, SARS-CoV-2 was detected on placental tissue also using an RNA in situ hybridization (ISH), which enabled the detection of the SARS-CoV-2 spike protein mRNA using a V-nCoV2019-S probe [26]. In this small series, the RNAscope probe detected positive staining for COVID-19 viral RNA in the infected tissues but not in the uninfected placentas [25]. A case of miscarriage during the second trimester of pregnancy in a woman with COVID-19 appeared to be related to placental infection with SARS-CoV-2 and was supported by virologic findings in the placenta. A placental swab was positive for SARS-CoV-2, and at histology, mixed inflammatory infiltrates composed of neutrophils and monocytes in the sub chorial space, intervillous fibrin deposition, and funisitis were demonstrated [27].

Recently, Shanes et al. evaluated 16 placentas from patients with SARS-CoV-2 to investigate the impact of COVID-19 infection on histology. In this study, 15 women had live birth in the third trimester; 1 delivered in the second trimester after intrauterine fetal demise. Compared to controls, third trimester placentas in patients affected with COVID-19 more significantly showed at least one feature of maternal vascular malperfusion, particularly abnormal or injured maternal vessels, and intervillous thrombi. Rates of acute and chronic inflammation were not increased. The authors stated that the pattern of placental injury reflected abnormalities in oxygenation within the intervillous space associated with adverse perinatal outcomes. However, none of the placentas in the study were tested for SARS-CoV-2 viral RNA or proteins [28].

Our report has no negligible limitations: We performed only qPCR for detecting the presence of SARS-CoV-2 in three different placental localizations by duplicate samples, but the placental swab was not repeated. Immunostaining and ISH for SARS-CoV-2 are not available in our institution; these methods allow visualizing the virus directly by evaluating the presence of the SARS-CoV-2 proteins as the molecular target while still retaining the tissue morphology, a feature that is lost with other methods, such as qPCR. Additionally, a qPCR evaluation on the chorionic villi scraped from the membranes and digested by enzymes (i.e., trypsin) was not done, as previously described by other authors [29]. Umbilical cord blood, amniotic and gastric fluid, anal swabs, or stools from the neonates can be theoretically collected to optimize the detection of viral infection. We did not collect and test these samples with qPCR according to our hospital protocol related to management of pregnant patients with suspected or confirmed COVID-19. Lastly, evaluation of the presence SARS-CoV-2 RNA in maternal milk was not possible as the woman decided to inhibit lactation onset one day after the caesarean section.

Overall, clinical reports on maternal and neonatal outcomes of pregnant women with SARS-CoV-2 infection remain limited. An ongoing multicenter, retrospective, cohort study (NCT04319016) is evaluating pregnancy and perinatal outcomes of pregnant women with COVID-19. All consecutive hospitalized and outpatient pregnant women with laboratory-confirmed 2019-n-CoV are being considered eligible for the study analysis; clinical symptoms or signs, laboratory findings, and maternal and perinatal outcomes are being collected [30].

## 4. Conclusions

Clinical data on maternal and neonatal outcomes of pregnant women with SARS-CoV-2 infection remain limited. This was a report of a positive placental swab for SARS-CoV-2 in an asymptomatic woman in the third trimester of pregnancy with a positive rhino-pharyngeal swab for COVID-19, who underwent an urgent caesarian section for obstetric indications. We are aware that our study does not have a design and a methodology suitable to definitively support a causal relationship between COVID-19 and placental infection or fetal transmission, similarly to other studies on the same topic published in the current literature. 

New studies will draw a definitive answer on the impact of COVID-19 on pregnancy, and in particular, on placental mechanisms that could be involved in response to maternal infection. 

## Supplementary Materials

The following are available online at https://www.mdpi.com/1010-660X/56/6/306/s1, Figure S1: Reverse-transcriptase quantitative PCR amplification curves and high-resolution melting curves of the amplification products for additional technical replicate of sample obtained in the placental fetal surface proximity to the umbilical cord.
