# Supplementary figures and images for: Report of Positive Placental Swabs for SARS-CoV-2 in an Asymptomatic Pregnant Woman with COVID-19
Source: Medicina (Kaunas). 2020 Jun 22;56(6):306. doi: 10.3390/medicina56060306 (PMC7353888; doi:10.3390/medicina56060306)

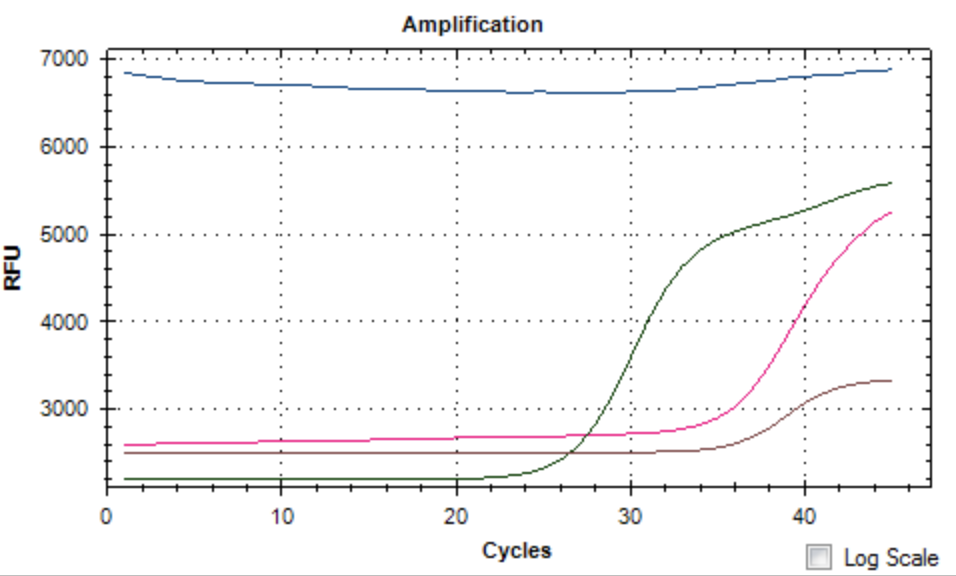

Supplement: Supplementary file 1 [file medicina-56-00306-s001.zip › medicina-801666-supplementary.png]
